# Mixed exposure to lead, methylmercury, and cadmium aggravates spatial memory deficits via dopamine signaling pathways in the mouse hippocampus

**DOI:** 10.3389/fpubh.2026.1801968

**Published:** 2026-03-25

**Authors:** Haesoo Kim, Daeun Lee, Sarita Pyatha, Kisok Kim

**Affiliations:** College of Pharmacy, Keimyung University, Daegu, Republic of Korea

**Keywords:** cadmium, dopamine, hippocampus, lead, methylmercury, neurotoxicity

## Abstract

**Background:**

Neurobehavioral disorders due to mixed exposure to toxic metals have been detected in many populations worldwide. This study investigates the impact of mixed-metal exposure on cognitive function and dopaminergic activity in mice.

**Methods:**

Spatial learning and memory were evaluated using the Morris water maze test. The expression of genes and proteins associated with dopaminergic neurotransmission in mice exposed to lead (Pb), methylmercury (MeHg), and cadmium (Cd) was examined in parallel. Male C57BL/6 mice (7 weeks old) were exposed to Pb (25 mg/L), MeHg (10 mg/L), Cd (15 mg/L), or a mixture (25 mg/L Pb + 10 mg/L MeHg + 15 mg/L Cd) in drinking water for 28 days.

**Results:**

Our results indicate significant deficits in cognitive function among metal-exposed groups, with the most pronounced impairments occurring in the mixed exposure group. In mice exposed to the metal mixture, dopamine levels in the hippocampus were significantly lower than those in control mice or, notably, in mice in the single-metal exposure groups. These findings were corroborated by real-time polymerase chain reaction and western blot analyses, which demonstrated altered expression of key dopaminergic markers, including tyrosine hydroxylase, dopamine transporters, and dopamine receptors.

**Conclusion:**

Our study underscores the detrimental effects of mixed-metal exposure on both cognitive function and dopaminergic signaling, highlighting the need for stringent environmental regulations to prevent or mitigate mixed exposure to toxic metals.

## Introduction

Lead (Pb), methylmercury (MeHg), and cadmium (Cd) are widely distributed in various forms and combinations in both living and working environments. Pb is distributed through industrial emissions and legacy sources such as lead-based paints and leaded gasoline (1), while MeHg bioaccumulates in aquatic food chains with fish consumption as the main human exposure route (2), and Cd is released from mining, smelting, and agricultural activities (3). Industrial activities including mining, smelting, battery manufacturing, and coal combustion are major sources of environmental Pb, MeHg, and Cd contamination (4, 5). These metals are released into air, water, and soil, leading to widespread human exposure through multiple pathways including inhalation, ingestion of contaminated food and water, and dermal contact (6). Chronic exposure to these metals through food, drinking water, and ambient air results in their accumulation in the body and may lead to health issues or diseases (7–9). The World Health Organization (WHO) and the Agency for Toxic Substances and Disease Registry (ATSDR) have classified these metals as hazardous environmental toxins that require attention (7–10). Because these metals typically occur in combination, exposure is often to a metal mixture rather than to a single metal, highlighting the need for research into the effects of combined exposure (11). Moreover, these metals have been detected in the blood and urine of populations worldwide (12, 13), such that a determination of the toxicological effects of combined exposure has become particularly important. However, limited research has examined the combined effects of Pb, MeHg, and Cd on hippocampal dopaminergic signaling and its relationship to cognitive impairment, representing a critical knowledge gap in understanding mixed metal neurotoxicity.

Pb exposure disrupts synaptic signaling, interferes with calcium-dependent neuronal processes, and induces neurotoxicity, which can have deleterious effects on the hippocampus, a brain region critical for learning and memory (14). MeHg exposure has been demonstrated to induce oxidative stress, mitochondrial dysfunction, and the inhibition of critical neuronal cells (15). Cd crosses the blood–brain barrier, accumulates in the brain, and induces oxidative stress and neuroinflammation, thereby impairing cognitive function, including in the hippocampus (16). The toxicity of Pb, MeHg, and Cd is amplified by their shared properties, such as environmental persistence, bioaccumulation in organisms, and biomagnification through food chains. Moreover, simultaneous exposure to Pb, MeHg, and Cd has been causally linked to neurotoxicity (17, 18). Previous studies reported the accumulation of all three metals in the brain through various pathways, resulting in central nervous system dysfunction (19–24). Specifically, movement disorders, behavioral and cognitive decline, and memory and learning disorders linked to metal exposure have been attributed to an impaired dopamine system (20, 25–29). Therefore, a potential hypothesis for this study is that combined exposure to Pb, MeHg, and Cd synergistically intensifies neurotoxic effects on the dopamine system and hippocampal function, leading to more severe cognitive and behavioral deficits.

The dopamine system is widely distributed throughout the central and peripheral nervous systems and plays important roles in reward, cognition, memory, and learning functions (30, 31). Decreased dopamine levels in the hippocampus and dysfunctional hippocampal dopamine receptors lead to an imbalance in the dopamine system and thus neurodegenerative diseases such as Alzheimer’s disease (32). The accumulation of heavy metals (e.g., Pb, MeHg, or Cd) in the brain causes damage to the dopamine system (33–36); the additive or synergistic effects of simultaneous heavy-metal exposure have been identified (37, 38).

Previous studies regarding the effects of heavy metal co-exposure in the striatum showed that exposure to Pb, MeHg, and Cd causes dysfunction within the striatal dopamine system (39). However, little is known about the effects of combined exposure to these metals on dopaminergic neurotransmission in the hippocampus, a crucial region of the brain involved in learning and memory. Therefore, this study examined the neurobehavioral consequences of mixed exposure to Pb, MeHg, and Cd by assessing learning and memory in mice. The underlying mechanisms were investigated by analyzing dopaminergic signaling in the hippocampus. Our findings provide novel mechanistic insights into the synergistic neurotoxic effects of mixed heavy metal exposure on hippocampal dopaminergic signaling and cognitive function.

## Materials and methods

### Animals and treatment

This study employed a randomized controlled experimental design to investigate the neurobehavioral effects of single and mixed heavy metal exposure in mice. Male C57BL/6 mice (*n* = 50, 7 weeks old, weighing 22.0 ± 0.5 g) were obtained from Samtako Bio Korea (Osan, Korea) after approval for animal experimentation from the Animal Experiment Ethics Committee at Keimyung University (approval no.: KM2022-002). After a 1-week acclimation period, the mice were randomly divided into five groups (*n* = 10 per group): water-treated control, Pb-treated (25 mg/L), MeHg-treated (10 mg/L), Cd-treated (15 mg/L), and metal mixture–treated (Pb + MeHg + Cd; abbreviated as Mix). All mice (*n* = 10/group) underwent behavioral testing. For biochemical analyses, hippocampal tissues from 5 randomly selected mice per group were analyzed by ELISA, while tissues from the remaining 5 mice were analyzed by real-time PCR and Western blot. The metal concentrations used in this study were based on previous research on the neurotoxic effects of Pb, MeHg, and Cd (40–42). The doses in this study were designed to simulate environmentally relevant, low-to-moderate exposure levels that could occur in contaminated environments. They were intended to produce detectable neurobehavioral and biochemical effects without inducing overt systemic toxicity. The metal-treated mice received Pb, MeHg, and Cd in the form of lead (II) acetate trihydrate (Pb(CH_3_COO)_2_·3H_2_O; ACS grade; Sigma-Aldrich, St. Louis, MO, USA), methylmercury chloride (CH_3_HgCl; analytical reagent grade; Sigma-Aldrich, St. Louis, MO, USA), and cadmium chloride (CdCl_2_; ACS grade; Sigma-Aldrich, St. Louis, MO, USA), respectively, which were added to their drinking water for 4 weeks. The metal solutions were prepared in distilled water, and both their solubility and stability were confirmed. Mice in the water-treated control group were given distilled deionized water. The animals were housed under a 12-h light/dark cycle at a temperature of 20–22 °C and relative humidity of 55 ± 5%; they received sufficient water and food (RodFeed, DBL Co., Umsung, Korea). Mice were group-housed (5 mice per cage, 2 cages per treatment group). Each cage was provided with a single water bottle, and water consumption was measured per cage and normalized to per-mouse estimates by dividing total cage consumption by the number of mice per cage. Food and water were provided ad libitum. The experiments were conducted in accordance with NIH guidelines for the Care and Use of Laboratory Animals, as well as the ARRIVE guidelines for reporting animal research. Food and water intake and body weight were measured once per week. The mice were euthanized using carbon dioxide (CO_2_) on day 29. The hippocampus was then dissected from the brain of each mouse and stored at −80 °C until it could be used in experiments.

### Morris water maze test

**Figure 1:**
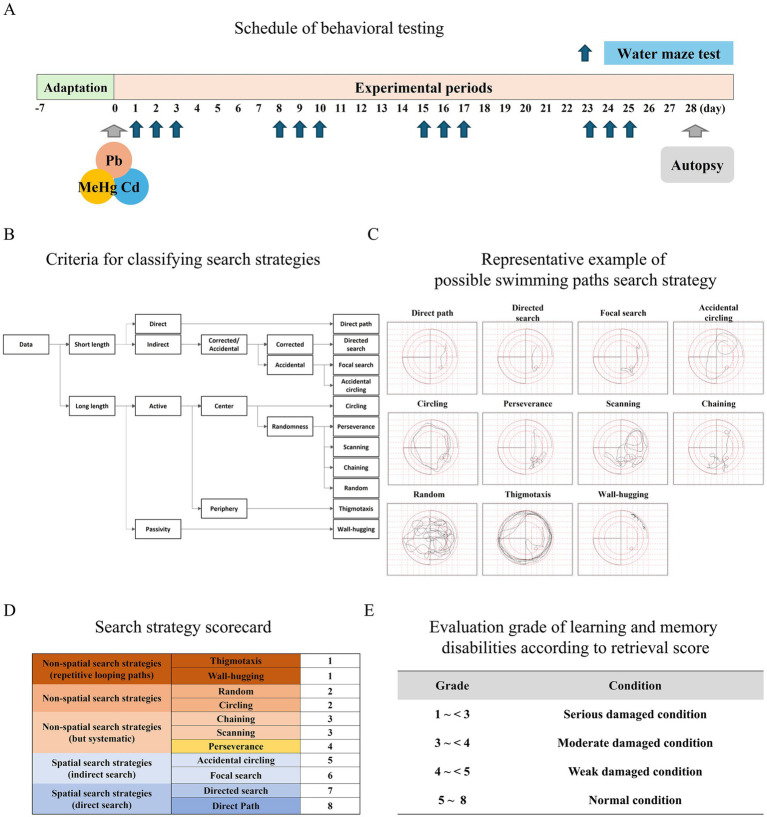
Experimental design and Morris water maze (MWM) platform search strategy classification during task learning. **(A)** Schedule of the mouse MWM test. **(B)** Criteria for classifying the search strategies; binary choice tree with modifications based on the binary decision as described by Illouz et al. (43) and Rai et al. (44). **(C)** Possible swimming paths in the search strategy; the swimming strategies utilized by mice in the MWM are sorted from highly cognitive to non-cognitive as follows (from top-left to bottom-right): direct, directed search, focal search, accidental circling, circling, perseverance, scanning, chaining, random, thigmotaxis, and wall-hugging. **(D)** Scorecard of the search strategy; classification and score according to the MWM search strategy. **(E)** Evaluation grade of learning and memory deficits according to the retrieval score.

Spatial learning and memory in the mice were evaluated using a Morris water maze (MWM) test, as described in a previous study (11). Before the experiment, all animals were acclimatized to the experimental conditions and environment for a minimum of 30 min. The MWM tests were conducted under lighting conditions of 20 lux; the water temperature was maintained at 27 ± 0.5 °C, and the water was made opaque using non-toxic white paint. The pool (120-cm diameter, 30-cm height) was divided into four quadrants in clockwise order: quadrant 1 (northeast), quadrant 2 (northwest), quadrant 3 (southwest), and quadrant 4 (southeast). A 9.5-cm-diameter platform was positioned in the southeast quadrant of a circular pool filled with tap water until the platform was submerged 0.5 cm below the water surface, 25 cm from the pool wall; this position was maintained throughout the experiment. The mice underwent training in a water-filled pool. The MWM testing protocol consisted of an initial 2-day adaptation phase (visible platform, 2 trials/day), followed by a 12-day hidden platform testing phase conducted over 4 consecutive weeks (3 days of testing per week, 2 trials/day, with 4-day rest periods between testing weeks). This resulted in a total of 12 testing days (Days 1, 2, 3, 8, 9, 10, 15, 16, 17, 22, 23, 24) over the 4-week exposure period (Figure 1A). During the first adaptation trial, the mice were placed in the northeast quadrant; in subsequent trials, they were randomly placed in either the northwest or southwest quadrant. In the actual experiment, the mice were placed in quadrant 1. Prior to testing, the mice were trained to find a visible platform, both in freshly prepared water and in water without added color. After the adaptation test using the visible platform, the mice were tested using the hidden platform as the MWM task. Data regarding the relevant indicators were recorded during the training and testing phases using DigBehv analytical software and a video tracking system (Smart Video Tracking System-SMART 3.0, Panlab, Barcelona, Spain). The MWM results were analyzed by classifying the swimming paths of mice into 11 search strategies according to the methods described by Illouz et al. and Rai et al. (43, 44) (Figures 1B,C). Scores were then assigned to each classified spatial search strategy (Figure 1D). The strategies ‘direct path’ and ‘directed search’ were assigned 8 and 7 points; ‘focal search’ and ‘accidental circling’ 6 and 5 points; ‘perseverance’, ‘scanning’, and ‘chaining’ 4, 3, and 3 points, respectively; ‘circling’ and ‘random’ 2 points each; and ‘thigmotaxis’ and ‘wall-hugging’ 1 point each. The spatial learning and memory functions of the mice were assessed based on the sum of these scores (Figure 1E).

### ELISA

To analyze dopamine levels in hippocampal tissue, the hippocampus was quickly dissected from the brains of control and metal-exposed mice; dopamine levels were analyzed using a mouse dopamine ELISA kit (Cusabio, Carlsbad, CA, USA), in accordance with the manufacturer’s instructions. The absorbances of the samples were measured at a wavelength of 450 nm using a microplate reader (Infinite 200 pro, Tecan, Männedorf, Switzerland). The detection range and sensitivity of the assay are 5–1,000 pg./mL and 2.5 pg./mL, respectively.

### Real-time PCR

Total RNA was extracted from the hippocampus using the NucleoSpin RNA kit (Macherey-Nagel, Germany), in accordance with the manufacturer’s protocol, and quantified using a NanoDrop 2000 spectrophotometer (Thermo Scientific, Waltham, MA, USA). RNA from each sample was reverse-transcribed using the iScript cDNA synthesis kit (Bio-Rad, Hercules, CA, USA), as specified by the manufacturer. The resulting cDNA was characterized using the SsoAdvanced Universal SYBR Green Supermix kit (Bio-Rad) on a CFX96 real-time PCR system (Bio-Rad). The following genes were analyzed in this study: Th (NM_009377), Dat (NM_010020), Vmat2 (NM_172523), Drd1 (NM_010076), Drd2 (NM_010077), and, for normalization, the housekeeping gene *β*-actin (NM_007393) (Table 1). Cq data obtained from the PCR amplifications were analyzed using the CFX Manager Software (Bio-Rad) to obtain the expression levels of the amplified genes.

**Table 1:** Primer sequences (5′ to 3′) and NCBI references.

Gene	NCBI gene accession number	Sense	Antisense
*β*-Actin	NM_007393	GCAGGAGTACGATGAGTCCG	ACGCAGCTCAGTAACAGTCC
Th	NM_009377	GCACATTTGCCCAGTTCTCC	GTACACCGGCTGGTAGGTTT
Dat	NM_010020	TGCTCTCAGTCATCGGCTTC	CTCTGTTGAACTGCCCGAGA
Vmat2	NM_172523	ATGTGTTCCCGAAAGTGGCA	AAGTTGGGAGCGATGAGTCC
Drd1	NM_010076	TAAGCCACCGGAAGTGCTTT	AAGGACCCAAAGGGCCAAAA
Drd2	NM_010077	AGTGAACAGGCGGAGAATGG	TAGACCGTGGTGGGATGGAT

### Western blot

The hippocampus was homogenized in radioimmunoprecipitation assay buffer (Sigma-Aldrich, St. Louis, MO, USA) containing 1% protease inhibitor and phosphatase inhibitor cocktails. After centrifugation (20 min, 4 °C), the supernatant was collected and the protein concentration was estimated using the Bradford method, with bovine serum albumin as the standard. The proteins were separated by SDS-PAGE on a 10% polyacrylamide gel, then transferred to a nitrocellulose membrane. The following primary antibodies were used for immunoblotting: mouse anti-TH monoclonal antibody (1:150,000 dilution; Sigma-Aldrich, St. Louis, MO, USA), rat anti-DAT monoclonal antibody (1:500 dilution; Santa Cruz Biotechnology, Santa Cruz, CA, USA), mouse anti-VMAT2 monoclonal antibody (1:1,000 dilution; Santa Cruz Biotechnology), rabbit anti-DRD1 monoclonal antibody (1:1,000 dilution; Life Technologies), and mouse anti-DRD2 monoclonal antibody (1,1,000 dilution; Santa Cruz Biotechnology). Immunoreactive bands were visualized using an enhanced chemiluminescence western blotting detection reagent (Amersham Biosciences, Piscataway, NJ, USA) and by incubating the nitrocellulose membrane with goat horseradish peroxidase-conjugated secondary antibody (Santa Cruz Biotechnology). Band intensities were quantified using the ImageJ program (NIH, Bethesda, MD, USA). *β*-actin served as a loading control for immunoblotting.

### Statistical analysis

The search strategy scores, dopamine levels, and mRNA and protein expression levels of the metal-treated mice were compared with those of control mice using a one-way ANOVA, followed by Tukey’s post-hoc test for multiple comparisons. Data normality was assessed with the Shapiro–Wilk test to ensure suitability for parametric analysis. Sample sizes were *n* = 10 per group for behavioral tests and *n* = 5 per group for biochemical analyses. All measurements are presented as the mean ± standard deviation (SD). Statistical significance was defined as ^*^p < 0.05, ^**^p < 0.01, or ^***^p < 0.001. All statistical analyses were performed using SAS v. 9.4 statistical software (SAS Institute Inc., Cary, NC, USA).

## Results

### Body weight, total food intake, and drinking water volume

**Figure 2:**
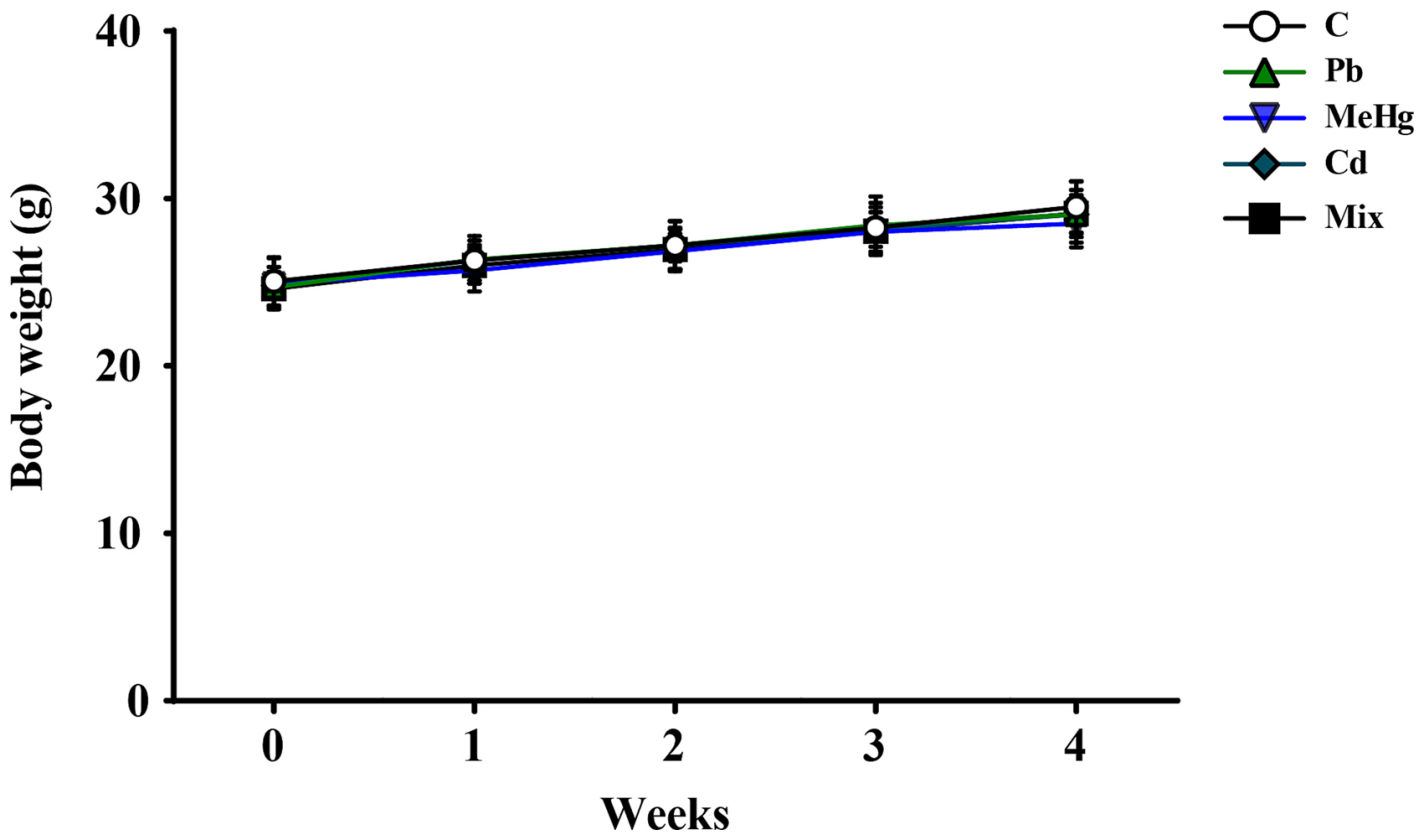
Changes in body weight during the experiment. Values are presented as mean ± SD (*n* = 10). C: Control, Pb: lead, MeHg: methylmercury, Cd: cadmium, Mix: Pb + MeHg + Cd.

During the study period, no overt signs of toxicity were observed in any of the mice. The body weight of mice in the metal-treated group was not significantly different from that of mice in the control group (Figure 2). There was also no significant difference in total food intake between the control and metal-treated groups (*F*_4,45_ = 1.36, *p* = 0.26; Supplementary Figure S1A). Average daily water consumption was 4.8 ± 0.6 mL/mouse/day and did not differ significantly among groups (*F*_4,45_ = 1.57, *p* = 0.20; Supplementary Figure S1B). Based on average body weight (27.1 ± 1.9 g) during the exposure period, the estimated daily doses were: Pb group (7.42 mg/kg/day), MeHg group (2.85 mg/kg/day), Cd group (3.76 mg/kg/day), and Mix group (7.42 mg/kg/day Pb + 2.85 mg/kg/day MeHg + 3.76 mg/kg/day Cd).

### Learning and memory function

**Figure 3:**
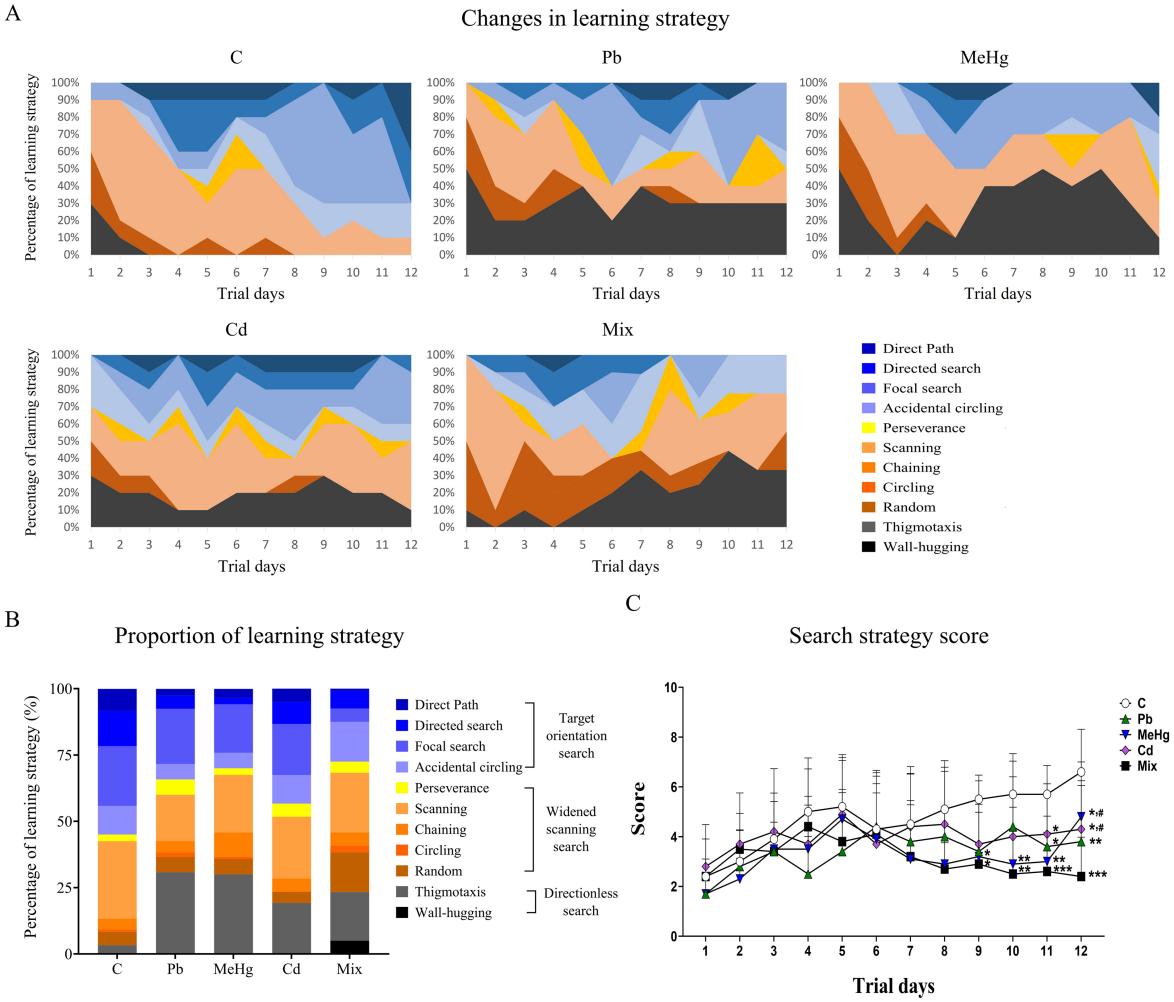
Spatial learning and memory function according to the search strategy classification and score in the MWM test. **(A)** Changes in learning strategy during the trial days in the control and metal-exposed groups. **(B)** Proportion of learning strategies during the 12-day experimental period. **(C)** Search strategy scores during the experiment. Each value is the mean ± SD (*n* = 10). ^*^p < 0.05 vs. control group; ^**^p < 0.01 vs. control group; ^***^p < 0.001 vs. control group; ^#^*p* < 0.05 vs. ix group. C: ontrol, Pb: ead, MeHg: ethylmercury, Cd: admium, Mix: Pb + MeHg + Cd.

The changes in the search strategies of the control and metal-treated mice during the MWM test are presented in Figure 3A. Over the course of the trial period, metal-treated mice showed a decrease in target-oriented search and an increase in directionless search compared with the control mice (Figure 3B). The search strategy score was significantly lower in the metal-treated groups than in the control group from the 9th trial day onwards, with statistically significant differences determined between all metal-treated groups and the control group on day 12 (*p* < 0.05, Figure 3C). Overall, the scores of the three single-metal-treated groups indicated weak to moderate damage. On day 12, the metal-mixture group exhibited the lowest search strategy score among all groups, suggesting severe cognitive impairment. This score was lower than those of the control and all single-metal–treated groups, with statistically significant differences observed relative to the MeHg and Cd groups (*p* < 0.05).

### Dopamine concentration

**Figure 4:**
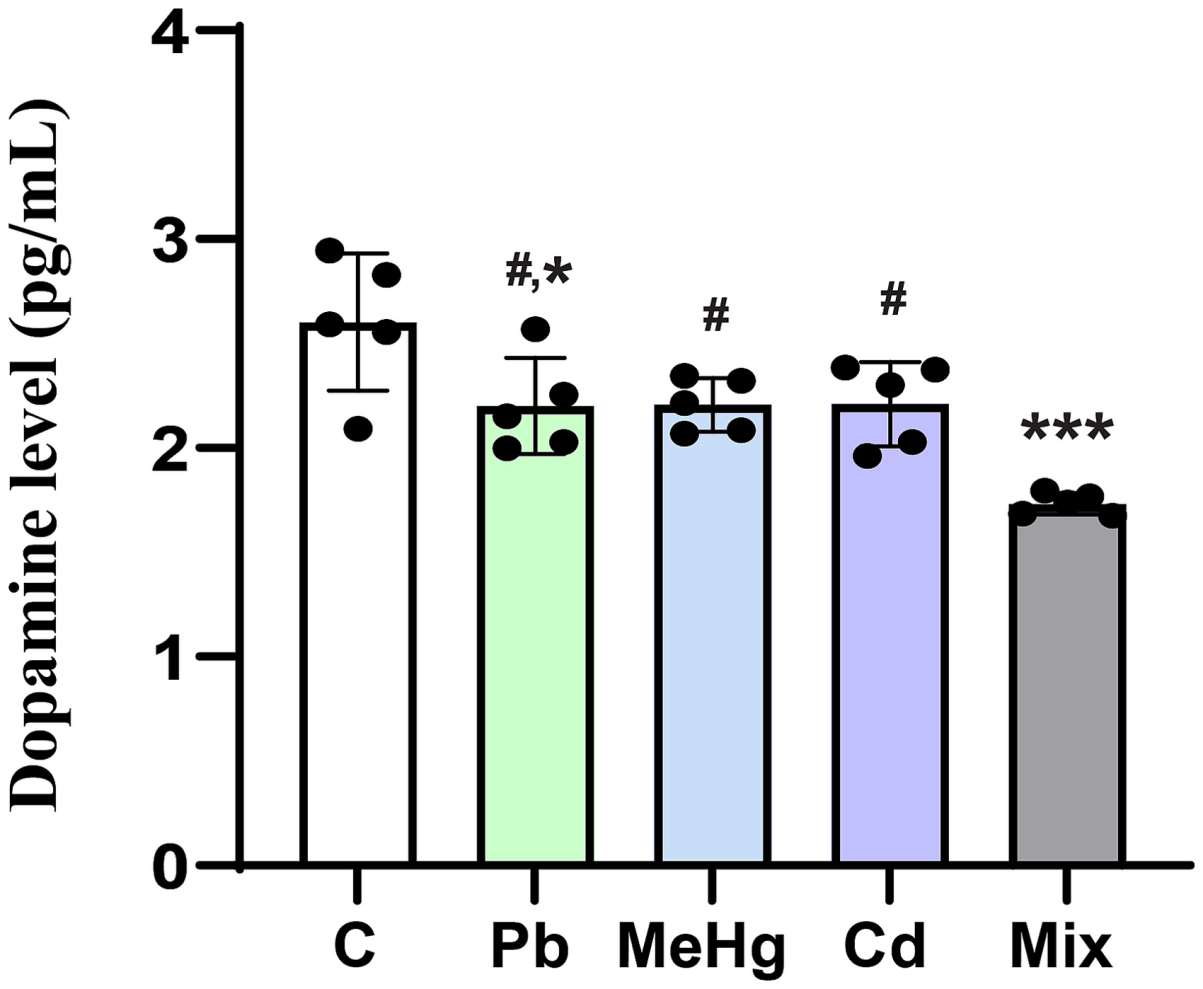
Effect of metal exposure on the dopamine level in the mouse hippocampus. Relative dopamine content in the hippocampus is expressed as the mean ± SD (*n* = 5). Individual values are shown as dots overlaid on bars representing the group mean ± SD. ^*^p < 0.05 vs. control group; ^***^p < 0.001 vs. control group; ^#^*p* < 0.05 vs. metal mixture (Mix) group.

Overall, single-metal exposure decreased dopamine levels in the mouse hippocampus. In the Pb-exposed group, dopamine levels were significantly lower than in the control group (*p* < 0.05, Figure 4). In the mixed-metal group, hippocampal dopamine levels were significantly lower than in either the control group (*p* < 0.001) or the single-metal-treated groups (*p* < 0.05).

### Gene and protein expression

**Figure 5:**
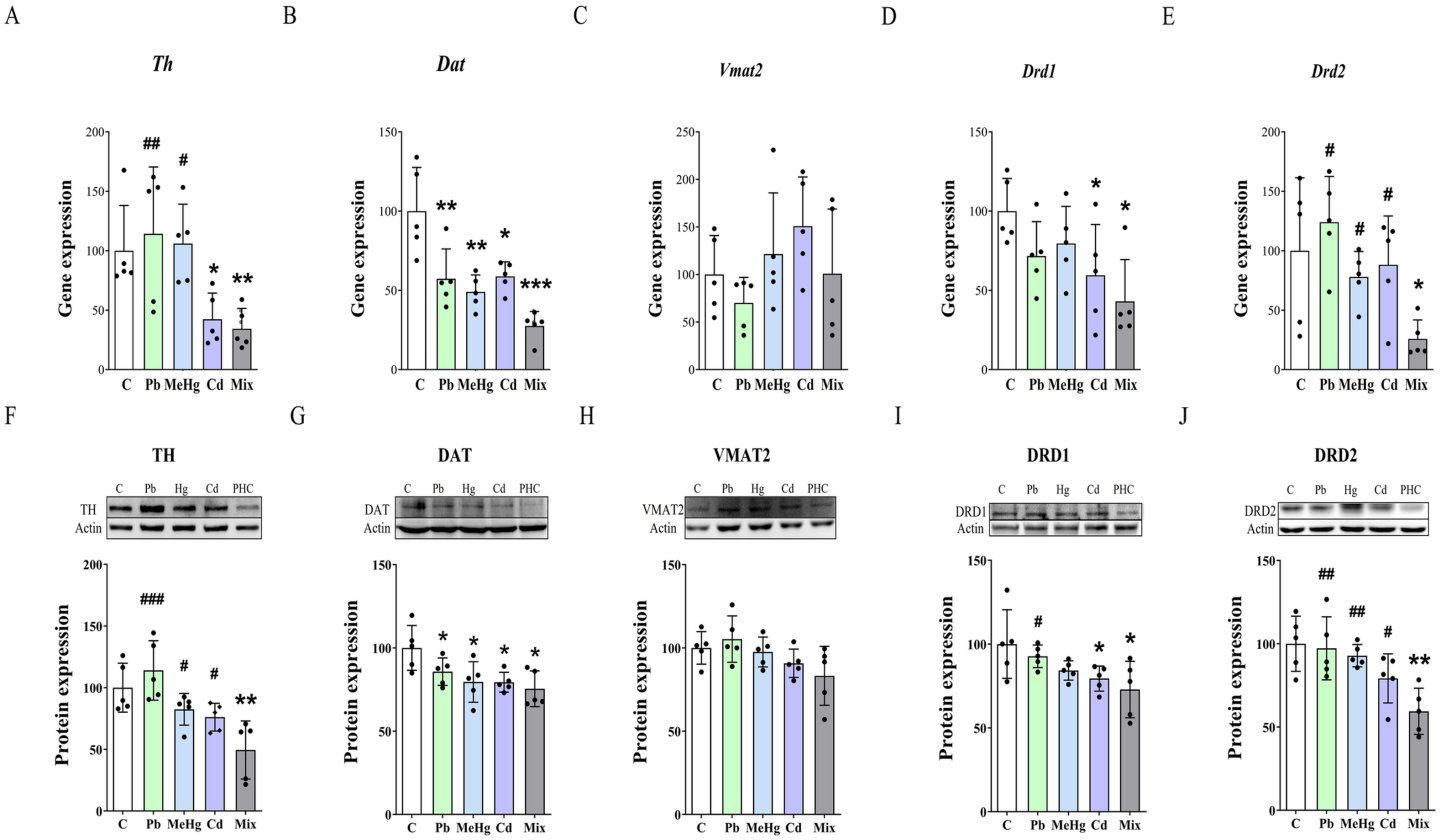
Effects of metal exposure on dopaminergic neurotransmission in the mouse hippocampus. **(A–E)** Relative mRNA expression levels of genes involved in dopaminergic neurotransmission in the hippocampus. **(F–J)** Representative western blots and relative expression levels of key proteins involved in dopaminergic neurotransmission. Data are presented as mean ± SD (*n* = 5), with individual values shown as dots overlaid on bars representing the group mean ± SD. ^*^p < 0.05 vs. control group; ^**^p < 0.01 vs. control group; ^#^*p* < 0.05 vs. metal mixture (Mix) group. ^##^*p* < 0.01 vs. Mix group; ^###^*p* < 0.001 vs. Mix group.

In the group exposed to the metal mixture, Th gene expression was significantly lower than in the control group and in the groups exposed to Pb or MeHg alone (*p* < 0.05 or *p* < 0.01, Figure 5A). At the protein level, TH expression in the mixed-metal group was significantly lower than in the control group (*p* < 0.01) and in all single-metal exposure groups (*p* < 0.05, Figure 5F). Expression of the DAT gene and of DAT protein was significantly lower in all metal-treated groups than in the control group (*p* < 0.05) (Figures 5B,G), whereas VMAT2 expression in all metal-treated groups did not significantly differ from that in the control group (Figures 5C,H). The expression levels of the DRD1 and DRD2 genes and their respective proteins were significantly lower in the mixed-metal group than in the control group (*p* < 0.01 or *p* < 0.05) (Figures 5D,E,I,J). DRD2 expression was significantly lower in the mixed-metal group than in the single-metal-treated groups (*p* < 0.01 or *p* < 0.05).

## Discussion

This study showed that exposure to a mixture of Pb, MeHg, and Cd impairs spatial learning and memory ability in mice, as measured using the MWM test. The MWM test, in which learning and memory are evaluated based on the ability to find a hidden platform, is widely used to assess neurocognitive disorders, such as Alzheimer’s disease, in rodent models (45). Brody and Holtzman proposed a method to analyze MWM search strategies and thereby assess spatial cognition in mice (46). Application of their method revealed a dose-dependent loss of spatial recognition in mice exposed to a mixture consisting of low levels of Pb, MeHg, and Cd. The authors concluded that an analysis of search strategies in the MWM test can be used to effectively assess the reductions in cognitive ability caused by a damaged hippocampus (17). The classification of swimming paths is more sensitive than the conventional method relying on escape latency (17, 47); therefore, it was used in this study to examine differences in spatial cognition among mice exposed to Pb, MeHg, and Cd. Our finding of lower spatial cognitive functions in mice exposed to a mixture of Pb, MeHg, and Cd than in either control mice or mice exposed to a single metal is consistent with previous studies (12, 17, 48), which showed that mixed-metal exposure was more damaging to the animals’ learning and memory abilities compared with single-metal exposure. These results demonstrate that simultaneous exposure to heavy metals, even at doses individually considered non-neurotoxic or less neurotoxic, can nonetheless cause serious neurobehavioral abnormalities.

Numerous studies have reported that the decline in learning and memory abilities characteristic of neurodegenerative diseases such as Alzheimer’s disease is closely related to dopaminergic neurotransmission in the hippocampal region (49–52). Exposures to Pb, MeHg, Cd, and other heavy metals are known to alter dopamine neurotransmission, leading to memory and learning impairments similar to those observed in Alzheimer’s disease (29, 53, 54). In this study, the dopamine content in the hippocampus decreased in all metal-treated groups, but the largest decrease was in the mixed-metal group, whether compared with the control group or the single-metal groups. These results are consistent with the more substantial decline in spatial learning and memory abilities in the mixed-exposure group than in the single-exposure groups.

The expression levels of proteins related to dopaminergic neurotransmission, including TH, DAT, DRD1, and DRD2, also decreased in all metal-treated groups compared with the control group, except for TH expression in the Pb-treated group. Additionally, the mixed-metal exposure group showed a significant decrease in TH and DRD2 protein expression compared with the single-metal exposure groups. Pb, MeHg, and Cd alter dopaminergic gene and protein expression through multiple mechanisms including transcriptional interference (disruption of transcription factors such as Nurr1 and Pitx3, altered DNA methylation), post-transcriptional effects (mRNA stability and translation efficiency), and activation of protein degradation pathways (proteasomal degradation and autophagy) (55–58). In addition, mixed metal exposure produces synergistic neurotoxic effects through concurrent transcriptional interference, depletion of cellular antioxidant defenses, and competition for metal-binding proteins (59). The downregulation of TH likely reduces dopamine synthesis, while decreased DRD2 expression impairs receptor-mediated signaling, both of which contribute to the memory deficits observed in this study. Multiple studies emphasize dopamine’s critical role in maintaining hippocampal long-term potentiation (LTP) through dopamine receptor regulation (31). Consequently, dopaminergic modulation in the hippocampus appears to be a key factor in learning and memory function. Beyond the observed changes in dopaminergic marker expression, mixed heavy metal exposure disrupts multiple biological mechanisms including PKC and PI3K-AKT signaling pathways, impairs synaptic plasticity through LTP suppression, induces oxidative stress and mitochondrial dysfunction, triggers neuronal apoptosis and dendritic atrophy, and inhibits hippocampal neurogenesis, collectively contributing to the spatial learning and memory deficits observed in our study (37, 39, 60, 61). The decreased expression of TH, DAT, and dopamine receptors, all of which are key components of the dopaminergic neurotransmission system, has also been reported in Alzheimer’s disease (49, 62–65).

The adverse effects of metal exposure on the hippocampal dopamine system are complex and are determined by the specific combination and chemical properties of the metals as well as the route and duration of exposure. The downregulation of TH, DAT, and dopamine receptor proteins in mice exposed to Pb, MeHg, and Cd disrupted dopamine synthesis and homeostasis, leading to the observed impairments in learning and memory. Interestingly, exposure to Pb, MeHg, and Cd did not affect the expression of VMAT2, which plays a role in transporting dopamine into synaptic vesicles. These results suggest that the neurobehavioral abnormalities caused by hazardous metals such as Pb, MeHg, and Cd are closely related to dysfunction of the hippocampal dopamine system at the levels of dopamine synthesis, transport, and reception within the hippocampus. The greater overall toxicity of a mixture of metals, compared with any single metal, may reflect synergistic, additive, or antagonistic effects depending on the type of interaction (66, 67). In this study, larger decreases in the expression of TH and DRD2 occurred in response to mixed-metal exposure than to single-metal exposure, suggesting that neurotoxic effects on TH and DRD2 lead to impairments in learning and memory ability.

One of the main limitations of this study is the absence of direct measurements of metal concentrations accumulated in the hippocampus; thus, future studies assessing metal levels in this region could strengthen our findings by directly linking exposure to tissue burden and neurotoxic effects. Another limitation of this study is the use of drinking water as the exposure route for MeHg, which differs from the primary human exposure pathway via fish consumption. While this approach ensured controlled dosing, future studies could explore dietary administration to better mimic real-world MeHg exposure. Conversely, a key strength of this study lies in the fact that, unlike many studies focusing on single-metal effects or striatal dopamine, it examines the effects of mixed-metal exposure on hippocampal dopamine, a less-explored area with implications for neurodegenerative diseases such as Alzheimer’s. Additionally, for translational relevance, this study employed an experimental design that reflects subchronic low-level environmental exposure in young adult humans.

In the hippocampus, multiple neurotransmitters, including glutamate, acetylcholine, and serotonin, play critical roles in learning and memory processes. While dopamine is widely recognized as a key regulator of motivation and cognition across the brain, its specific contributions within the hippocampus remain insufficiently characterized, prompting this study to focus primarily on the dopaminergic pathway. Given this, future research should pursue a comprehensive analysis of additional neurotransmission systems, such as the cholinergic and glutamatergic pathways, which may also be influenced by combined metal exposure. Moreover, further studies are needed to investigate potential interactions between these pathways and dopaminergic signaling, offering a more integrated perspective on their collective effects on cognitive function.

The findings of this study have significant implications for human health, particularly for populations exposed to Pb, MeHg, and Cd in contaminated environments such as industrial regions, mining communities, or areas with inadequate water treatment. Our results suggest that mixed-metal exposure may pose a greater risk to cognitive function than single-metal exposure due to synergistic effects on the hippocampal dopaminergic system. This is particularly relevant for vulnerable populations, such as children, pregnant women, and workers in industries like battery manufacturing or fish processing, where combined exposure to Pb, MeHg, and Cd is common. These findings underscore the need for stringent environmental regulations and biomonitoring programs to mitigate the neurotoxic risks of mixed-metal exposure in these populations.

## Conclusion

Mixed exposure to Pb, MeHg, and Cd induced more severe learning and memory dysfunction than exposure to any single metal. The neurobehavioral deficit was associated with decreased dopamine levels in the hippocampus and reduced expression of key proteins involved in dopaminergic neurotransmission (TH, DAT, DRD1, DRD2). Notably, significantly greater reductions in dopamine content, as well as TH and DRD2 expression, occurred in mice simultaneously exposed to all three metals compared with any one metal alone, which aligns with the more severe cognitive impairment observed after mixed exposure. These findings underscore the importance of evaluating the neurotoxicity of mixed metal exposures because their effects may be greater than those predicted by individual metal assessments. Further research is needed to elucidate the mechanisms underlying the neurotoxicity of various heavy metal combinations.

## Data Availability

The raw data supporting the conclusions of this article will be made available by the authors, without undue reservation.
