# Supplementary material for: Mixed exposure to lead, methylmercury, and cadmium aggravates spatial memory deficits via dopamine signaling pathways in the mouse hippocampus
Source: Front Public Health. 2026 Mar 25;14:1801968. doi: 10.3389/fpubh.2026.1801968 (PMC13057470; doi:10.3389/fpubh.2026.1801968)
Supplement: Supplementary file 1 [file Data_sheet_1.docx]

**Supplementary Materials**

(Food and drinking water intake data, and original Western blot images)

**Article title:** Mixed Exposure to Lead, Methylmercury, and Cadmium Aggravates Spatial Memory Deficits via Dopamine Signaling Pathways in the Mouse Hippocampus


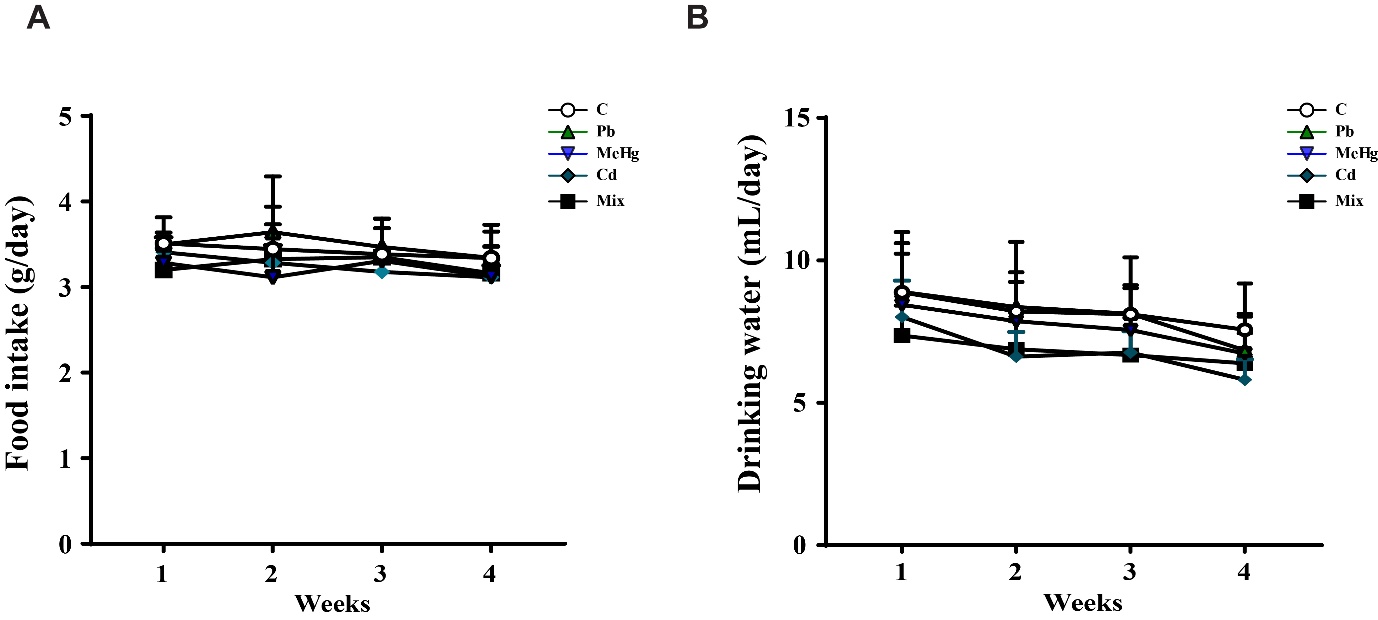


**Figure S1.** Changes in (A) food intake and (B) drinking water intake during the experimental period. Values are presented as the mean ± SD (n = 10). C, control; Pb, lead; MeHg, methylmercury; Cd, cadmium; Mix, Pb + MeHg + Cd.


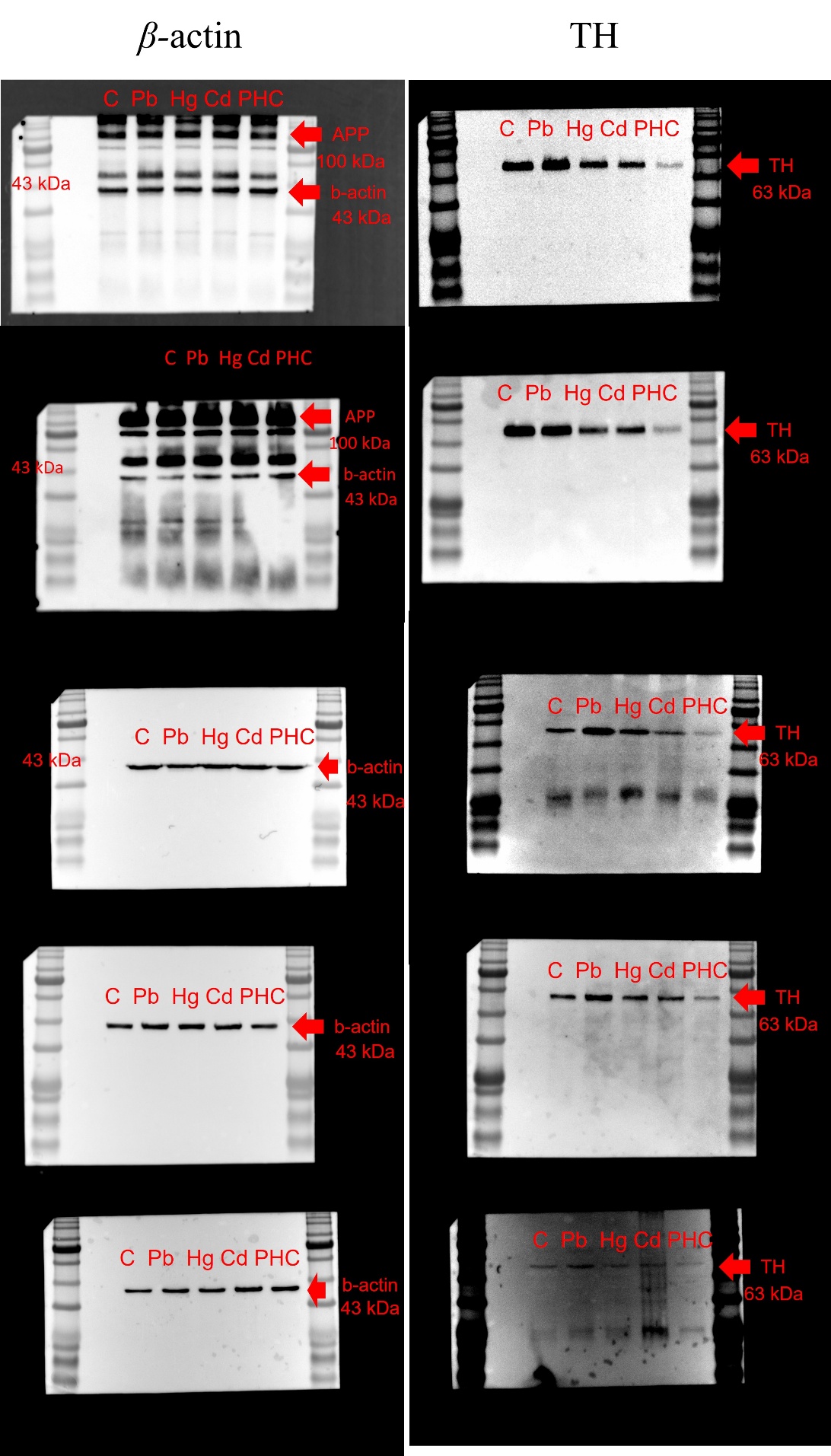


**Figure S2.** Original images of Western blotting data showing tyrosine hydroxylase (TH) and β-actin expression. C: control, Pb: lead, Hg: methylmercury, Cd: cadmium, PHC: Pb+Hg+Cd.


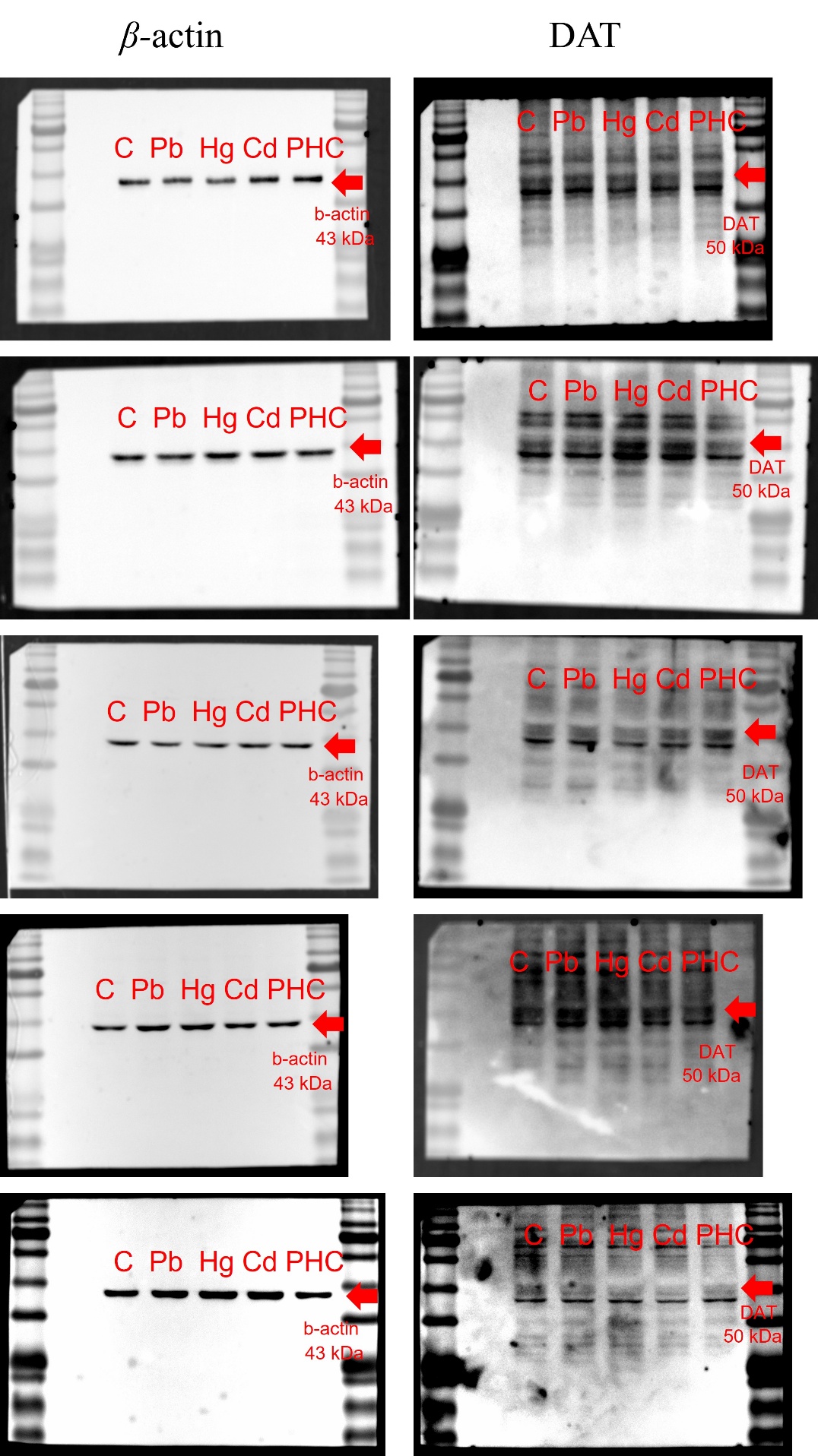


**Figure S3.** Original images of Western blotting data showing dopamine transporter (DAT) and β-actin expression. C: control, Pb: lead, Hg: methylmercury, Cd: cadmium, PHC: Pb+Hg+Cd.


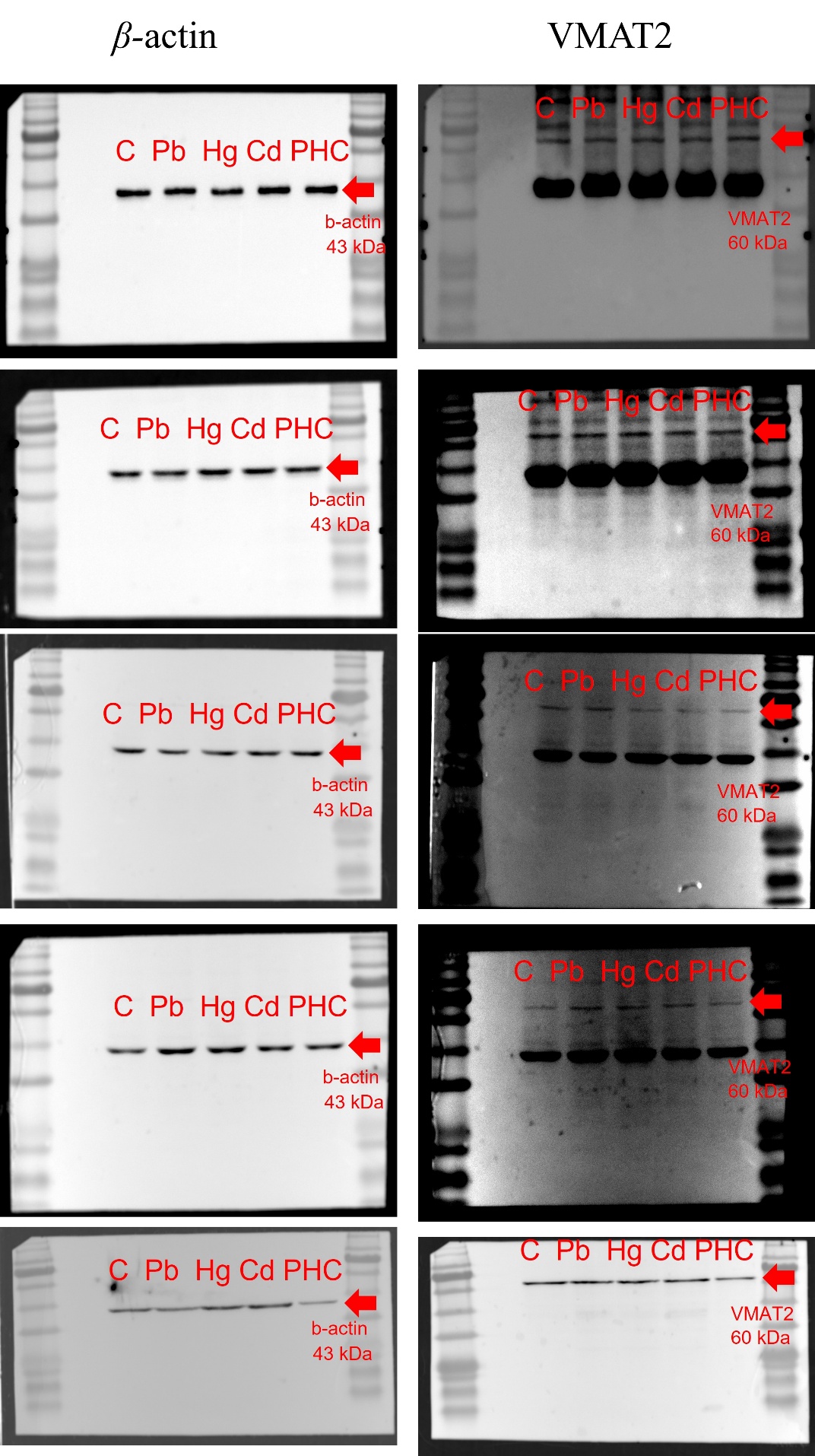


**Figure S4.** Original images of Western blotting data showing vesicular monoamine transporter 2 (VMAT2) and β-actin expression. C: control, Pb: lead, Hg: methylmercury, Cd: cadmium, PHC: Pb+Hg+Cd.


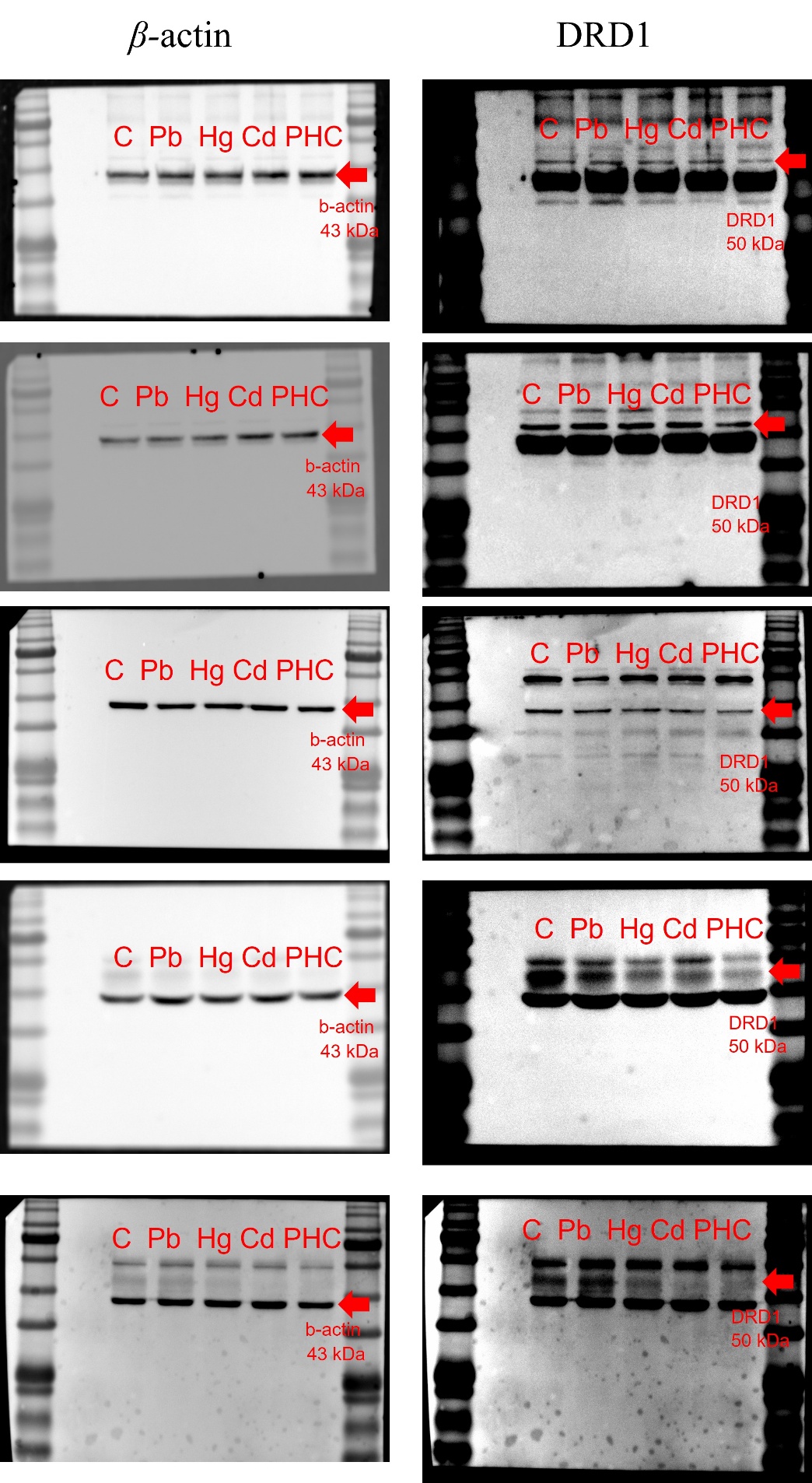


**Figure S5.** Original images of Western blotting data showing dopamine receptor 1 (DRD1) and β-actin expression. C: control, Pb: lead, Hg: methylmercury, Cd: cadmium, PHC: Pb+Hg+Cd.


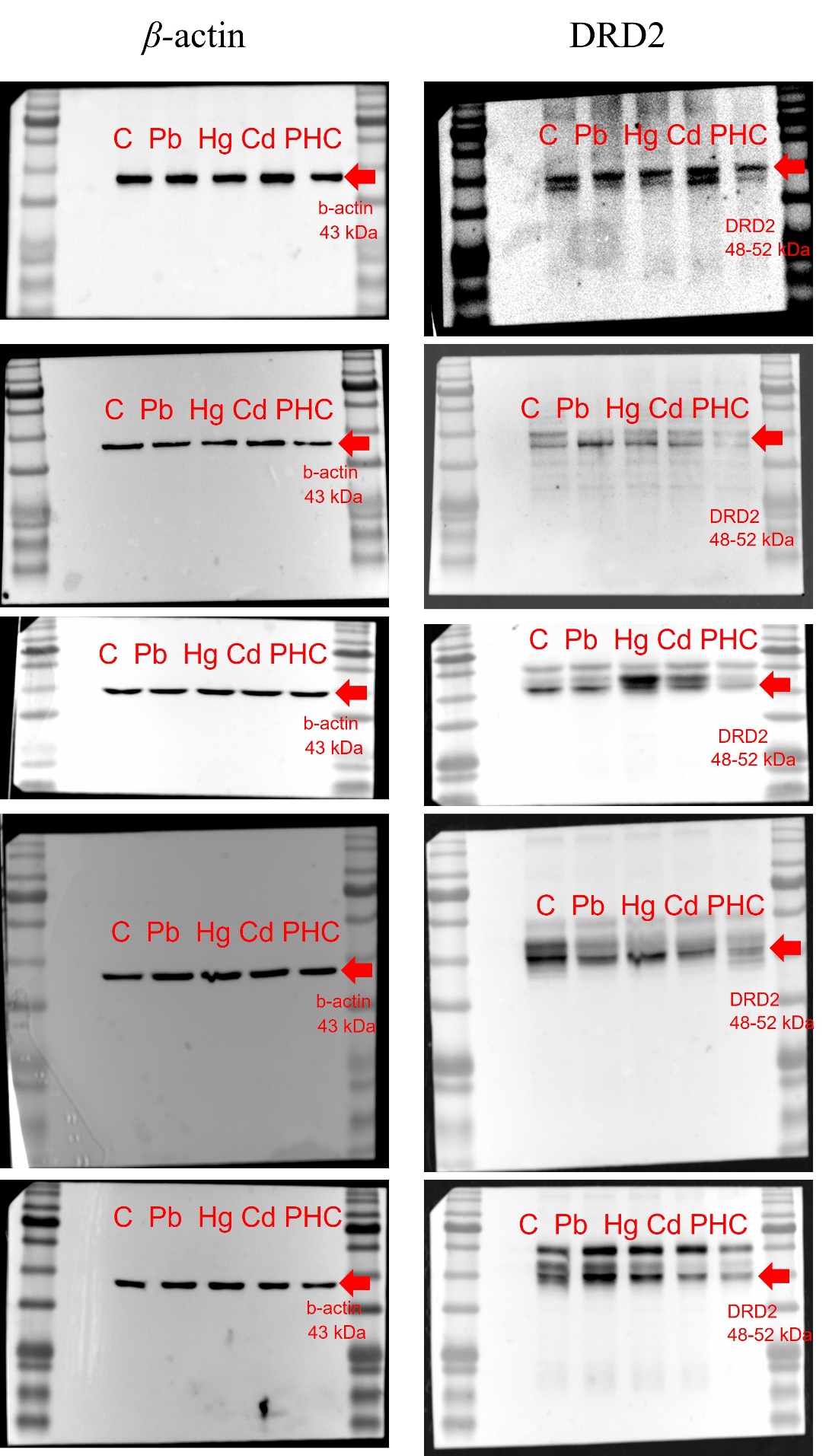


**Figure S6.** Original images of Western blotting data showing dopamine receptor 2 (DRD2) and β-actin expression. C: control, Pb: lead, Hg: methylmercury, Cd: cadmium, PHC: Pb+Hg+Cd.
